# Metabolic responses of microbial consortia to winery wastewater-associated inhibitors

**DOI:** 10.1007/s00253-026-13983-y

**Published:** 2026-08-03

**Authors:** Ana Baía, Alonso I. Arroyo-Escoto, Bilel Abdelkarim, Nuno Ramos, César Afonso, Gaetano R. Pesce, Maria C. Fernandes, Annabel Fernandes

**Affiliations:** 1 Fiber Materials and Environmental Technologies (FibEnTech-UBI), Universidade da Beira Interior, Covilhã, Portugal; 2 Department of Chemistry, Universidade da Beira Interior, Covilhã, Portugal; 3 Alentejo Biotechnology Center for Agriculture and Agro-Food (CEBAL), Polytechnic Institute of Beja (IPBeja), Beja, Portugal; 4 MED – Mediterranean Institute for Agriculture, Environment and Development & CHANGE – Global Change and Sustainability Institute, CEBAL- Alentejo Biotechnology Center for Agriculture and Agro-Food, Beja, Portugal; 5 MED – Mediterranean Institute for Agriculture, Environment and Development & CHANGE– Global Change and Sustainability Institute, Institute for Advanced Studies and Research (IIFA), University of Évora, Évora, Portugal; 6 Department of Agronomy, Food, Natural Resources, Animals and Environment—DAFNAE, University of Padua, Agripolis Campus, Legnaro, PD Italy

**Keywords:** Anaerobic consortia, Inhibitory compounds, Agro-industrial effluents, 16S rRNA gene sequencing, Metabolic resilience, Fumarate reduction

## Abstract

**Abstract:**

This study evaluates how non-conventional anaerobic microbial consortia from sanitary landfill leachate and mining wastewater respond to selected winery wastewater-associated inhibitors, including alcohols, carboxylic acids, and phenolic compounds. Batch assays were conducted in nutrient broth acetate–fumarate medium under single and combined inhibitory conditions. The enriched consortia were characterized by full-length 16S rRNA gene sequencing. Both consortia exhibited comparable growth rates under control conditions, but showed distinct growth dynamics, fumarate consumption, succinate and acetate profiles, and inhibitor consumption patterns. The landfill-derived consortium was dominated by *Escherichia* and displayed faster fumarate consumption under control and alcohol-supplemented conditions, indicating higher baseline substrate turnover. In contrast, the mining-derived consortium showed a more distributed taxonomic profile, dominated by *Proteus*, *Wolinella*, and *Escherichia*, and maintained fumarate consumption more consistently under carboxylic acid and phenolic stress. Alcohols had limited effects on growth and fumarate consumption, whereas carboxylic acids significantly reduced growth and altered metabolite profiles. Phenolic compounds exerted a strong inhibitory effect, particularly at high concentrations. Catechol and tyrosol showed only minor concentration changes, consistent with low apparent consumption under the tested anaerobic conditions. Under combined inhibitory conditions, both consortia maintained fumarate consumption and metabolite formation despite reduced growth, with the mining-derived consortium showing increased fumarate consumption and enhanced succinate production. Microbial source, enrichment history, community composition, and inhibitory conditions jointly shaped the resilience and metabolic performance of the anaerobic consortia. This work supports screening non-conventional microbial consortia for future anaerobic and bioelectrochemical treatment of chemically complex agro-industrial wastewater.

**Key points:**

• *Non-conventional anaerobic consortia maintained activity under inhibition*

• *Taxonomic composition was associated with distinct metabolic profiles*

• *Phenolics caused strong inhibition and low apparent anaerobic consumption*

**Supplementary Information:**

The online version contains supplementary material available at https://doi.org/10.1007/s00253-026-13983-y.

## Introduction

The increasing generation of agro-industrial wastewater poses significant environmental challenges due to its high organic load and complex chemical composition. Among these effluents, winery wastewater is characterized by marked seasonal and operational variability, with chemical oxygen demand (COD), alcohols, carboxylic acids, and phenolic compounds strongly depending on the production stage and cleaning practices (Akpor et al. 2014; Liu et al. 2021; Ngwenya et al. 2022). Ethanol is frequently one of the main contributors to the organic load and has been reported in the g L^−1^ range, whereas carboxylic acids are commonly present at concentrations ranging from tens to several hundreds of mg L^−1^ (Colin et al. 2005; Welz et al. 2016). Phenolic compounds usually occur at lower concentrations, but their inhibitory and recalcitrant character can interfere with microbial activity and increase the complexity of winery wastewater treatment (Jafary et al. 2019; Veeresh et al. 2005). Together, these compounds represent chemically and functionally distinct fractions of winery wastewater.

Treatment approaches for winery wastewater include conventional physicochemical and biological processes, as well as more advanced technologies such as electrochemical systems and membrane filtration, each with specific advantages and limitations (Akpor et al. 2014; Melchiors and Freire 2023). Electrochemical oxidation and membrane filtration can be effective for removing recalcitrant compounds, but their application may be constrained by energy demand and membrane fouling, respectively (Baía et al. 2022; Priyadarshini et al. 2022; El Rayess et al. 2024). Biological processes remain particularly relevant because they can respond to variations in organic loading and effluent composition, which are typical of winery operations (Bolzonella et al. 2019). Aerobic systems can achieve high COD removal, with efficiencies reported up to 98% under suitable organic loading rates, but they depend on efficient oxygen transfer and continuous aeration, increasing energy requirements and operating costs (Bolzonella et al. 2019). Anaerobic processes, in contrast, are attractive for high-strength agro-industrial effluents because they do not require aeration and can convert part of the organic load into reduced end-products or energy carriers, such as methane or hydrogen, depending on the reactor configuration and microbial metabolism involved (Bolzonella et al. 2019; Logan et al. 2008, 2023).

Although many aerobic bacteria can tolerate or degrade alcohols and carboxylic acids, as well as a wide range of aromatic compounds (Seo et al. 2009), anaerobic microbial consortia remain particularly relevant for high-strength and oxygen-limited effluents. In these systems, different microbial populations can contribute complementary functions, including fermentation, organic acid conversion, electron transfer reactions, and the transformation of inhibitory compounds (Harirchi et al. 2022; Logan et al. 2023). Such functional diversity can provide greater robustness than single-strain systems, especially when wastewater composition varies over time.

From a metabolic perspective, fermentative microorganisms can convert readily biodegradable substrates, such as alcohols and carboxylic acids, into intermediate metabolites, including volatile fatty acids, reduced end-products, and electron carriers (Harirchi et al. 2022). These intermediates may then be used by other microorganisms within the consortium, helping to maintain carbon conversion and electron balance under anaerobic conditions. In oxygen-free systems, this balance depends on alternative electron-accepting routes and on microbial interactions that prevent the accumulation of inhibitory intermediates. In winery wastewater, alcohols and carboxylic acids may enter anaerobic carbon fluxes, whereas phenolic and aromatic compounds represent more challenging substrates due to their inhibitory and recalcitrant nature. Evaluating how anaerobic consortia respond to these compound classes, individually and in combination, is therefore relevant for selecting microbial communities with improved tolerance under chemically complex conditions.

Despite the relevance of anaerobic processes for high-strength winery wastewater, most studies have focused on conventional inocula, such as activated sludge or anaerobic digester communities, with limited attention to microbial consortia originating from extreme or chemically stressed environments (Lauzurique et al. 2023; Ibáñez-López et al. 2023). Sanitary landfill leachate is typically characterized by a high organic load, recalcitrant organic matter, ammonium, humic substances, and reducing conditions, which may favor fermentative, syntrophic, and anaerobic organic matter-degrading microorganisms (Adhikari and Khanal 2015; Ishaq et al. 2023). Mining wastewater, in contrast, is commonly associated with low pH, metal exposure, sulfate-rich conditions, and limited organic carbon availability, selecting microbial communities exposed to chemical stress, redox imbalance, and metal- or sulfur-associated metabolism (Mendonça et al. 2003; Pattanaik et al. 2020).

These contrasting environmental pressures make such non-conventional sources relevant for exploring stress-tolerant anaerobic consortia. Nevertheless, their metabolic response to mixtures of inhibitory compounds associated with winery wastewater remains poorly explored. This limits the understanding of how microbial source and enrichment history shape metabolic resilience in non-conventional consortia.

Therefore, this study aimed to evaluate the tolerance and metabolic behavior of microbial consortia derived from landfill leachate and mining wastewater when exposed to selected alcohols, carboxylic acids, and phenolic compounds associated with winery wastewater. These compound groups were used as model representatives of biodegradable carbon sources, organic acid intermediates, and inhibitory/recalcitrant compounds. The experimental system was intended to isolate the effects of selected winery wastewater-associated inhibitors rather than to reproduce the full composition of real winery wastewater. By analyzing growth dynamics, fumarate consumption, succinate formation, acetate profiles, and the consumption patterns of selected inhibitors, this work examines key phenotypic and metabolic responses associated with anaerobic carbon conversion and inhibitor tolerance under defined conditions.

## Materials and methods

### Microbial consortia

Microbial consortia were obtained from two distinct environmental sources: mining wastewater collected from Mina de São Domingos (MSD) and sanitary landfill leachate (SLL) collected from the intermunicipal facility of Associação de Municípios do Alentejo Central, both located in Alentejo, Portugal. After collection, samples were stored under refrigeration to preserve microbial activity until use.

For enrichment, 5 mL of each wastewater sample was inoculated into 70 mL of nutrient broth acetate–fumarate (NBAF) medium, prepared as previously described (Huerta-Miranda et al. 2019). The medium contained 30 mM sodium acetate and 40 mM sodium fumarate, supplemented with 0.5 g L^−1^ yeast extract and 0.12 g L^−1^ cysteine. The NBAF medium was used as a controlled anaerobic basal medium to enrich the consortia and evaluate their metabolic activity under defined conditions. The acetate–fumarate composition provides a defined carbon-source/electron-acceptor system, in which acetate serves as a basal carbon and electron donor, while fumarate acts as a defined electron acceptor whose reduction to succinate can be followed as an indicator of anaerobic electron-acceptor use and redox-associated metabolism (Huerta-Miranda et al. 2019). This medium was not intended to reproduce the full composition or variability of real winery wastewater. Instead, it provided a reproducible background in which selected winery wastewater-associated inhibitors could be added and their effects on the two consortia compared. The acetate–fumarate system also provided metabolic readouts that may be useful for future screening of consortia for anaerobic or bioelectrochemical applications, although such systems were not tested in the present study (O’Brien and Malvankar 2016; Logan et al. 2008, 2023).

Anaerobic conditions were established by sparging the medium with an 80:20 (v/v) N_2_/CO_2_ gas mixture for 15 min before sterilization. The medium was then autoclaved at 121 °C for 20 min. Cultures were incubated at 36 °C under static conditions in a thermostatic cabinet. The enrichment procedure was maintained for 3 months through sequential batch transfers. Every 5 days, 5 mL of culture from the previous enrichment flask was transferred into 70 mL of fresh anaerobic NBAF medium, corresponding to an inoculum ratio of approximately 7% (v/v). This fixed transfer interval was used to allow repeated growth under acetate–fumarate conditions while maintaining a regular selective pressure during enrichment. Growth and metabolite profiles were not monitored across all enrichment cycles. Therefore, the procedure is described as an enrichment step under acetate–fumarate conditions, without implying demonstrated stability of community composition or metabolic performance during the enrichment period.

The microbial consortia used in this study are available from the corresponding authors upon reasonable request for research purposes. The authors are committed to providing access to these consortia to facilitate reproducibility and further research.

### Taxonomic characterization of the enriched consortia

The enriched SLL and MSD consortia used in the inhibition assays were submitted to an external sequencing service for bacterial taxonomic characterization based on the full-length 16S rRNA gene (Stab Vida Lda., Portugal). Total DNA was extracted using the Maxwell® RSC PureFood GMO and Authentication Kit, according to the manufacturer’s instructions. Full-length 16S rRNA gene amplification was performed using the universal primers 27 F (5′-AGAGTTTGATCMTGGCTCAG-3′) and 1492R (5′-CGGTTACCTTGTTACGACTT-3′), covering the V1–V9 hypervariable regions. Sequencing libraries were prepared using the 16S Barcoding Kit V14 (SQK-16S114.24; Oxford Nanopore Technologies) and sequenced on a MinION Mk1D platform using a Flongle Flow Cell. Basecalling was performed in Super Accuracy mode (SUP; version 5.2.0, 400 bps). Taxonomic classification was carried out using the wf-emu workflow provided by Oxford Nanopore Technologies, with a combined database composed of rrnDB v5.10 and NCBI 16S RefSeq. Read alignment was performed with minimap2, retaining up to 50 alignments per read, followed by resolution of multi-mapped reads using an Expectation–Maximization algorithm. To reduce the impact of false positives and sequencing-associated noise, taxa were retained only when their relative abundance was ≥ 0.01%. No isolation of individual strains was performed. Therefore, the inocula used in this study should be interpreted as enriched mixed microbial consortia rather than pure cultures. Because the analysis was based on 16S rRNA gene sequencing, fungal communities were not assessed.

### Fermentation experiments

Fermentation experiments were conducted in 150 mL anaerobic culture flasks, with a final working volume of approximately 80 mL, comprising inoculum and a controlled model medium containing selected winery wastewater-associated inhibitors. This medium consisted of 70 mL of enriched NBAF medium, as described in the “Microbial consortia” Section, supplemented with 1 mL of inhibitor solution. In each assay, the inoculum volume was adjusted to achieve an initial optical density (OD_600_) of approximately 0.4. To evaluate the effect of inhibitor concentration, each group of compounds, alcohols, carboxylic acids, and phenolics, was tested individually at two concentration levels, defined as low concentration (LC) and high concentration (HC), as detailed in Table 1.

**Table 1:** Composition of the inhibitor groups used in the fermentation experiments and the concentrations tested

Inhibitor group	Compound	Concentration (g L^−1^)	
		LC	HC
Alcohols	Ethanol	2.5	5.0
	Glycerol	0.16	0.32
	Methanol	0.0075	0.015
Carboxylic acids	L(+)-tartaric acid	0.26	0.53
	Lactic acid	0.18	0.35
	Acetic acid	0.33	0.66
	Succinic acid	0.04	0.08
Phenolics	Tyrosol	0.36	0.72
	Catechol	0.36	0.72

*LC* low concentration, *HC* high concentration

The selected compounds were chosen as representative model components of three chemically and functionally distinct fractions of winery wastewater. Ethanol, glycerol, and methanol were included as alcohols contributing to the biodegradable organic load, with ethanol often reported as one of the main contributors to winery wastewater COD (Colin et al. 2005; Welz et al. 2016). Tartaric, lactic, acetic, and succinic acids were selected as carboxylic acids commonly associated with grape composition, fermentation, and wastewater acidification (Welz et al. 2016). Tyrosol and catechol were used as model phenolic compounds because of the inhibitory and recalcitrant character of phenolic structures (Jafary et al. 2019; Veeresh et al. 2005; Boll et al. 2014). The LC levels were selected to fall within, or close to, concentration ranges reported for winery wastewater, whereas the HC levels were included as elevated-stress conditions to assess the tolerance limits of the enriched consortia (Colin et al. 2005; Welz et al. 2016; Zacharof 2017; Cañadas et al. 2022). Therefore, the experimental design was not intended to reproduce the full composition of real winery wastewater but to provide a controlled comparison of the effects of key winery wastewater-associated inhibitor groups. After testing each inhibitor group individually, a second set of experiments was conducted using a mixture of all inhibitor groups at their respective high concentrations to evaluate the response of the consortia to a combined inhibitory scenario.

Control assays were conducted for each consortium under identical experimental conditions without inhibitors, providing a baseline for comparison. Each experimental condition was performed in duplicate in two independent experimental runs, yielding four independent biological replicates in total. Each biological replicate corresponds to a separately inoculated anaerobic culture flask. Cultures were incubated under anaerobic conditions at 36 °C, as described in the “Microbial consortia” Section.

### Analytical methods and calculations

OD_600_ was measured using a UV–visible spectrophotometer (Thermo Scientific Evolution 201). For each experimental condition, OD_600_ measurements were blank-corrected using condition-specific abiotic blanks containing NBAF medium and the corresponding inhibitor solution, but no inoculum. These blanks were used to account for the optical background of the medium and inhibitor mixtures. Culture samples were gently homogenized before measurement. The linear range of OD_600_ measurements was verified using serial dilutions of representative cultures. When necessary, samples were diluted with sterile medium to remain within the linear measurement range, and the measured OD_600_ values were corrected by the corresponding dilution factor.

The concentrations of the target compounds monitored in the fermentation assays, including ethanol, glycerol, fumarate, succinic acid, acetic acid, lactic acid, tartaric acid, catechol, and tyrosol, were determined by high-performance liquid chromatography (HPLC) using a Shimadzu system equipped with a refractive index detector (RID-20A). Separation was performed using a Bio-Rad Aminex HPX-87H column under isocratic conditions with a 5 mM aqueous sulfuric acid solution as the mobile phase, at a flow rate of 0.6 mL min^−1^ and a column temperature of 50 °C. The injection volume was 5 µL. Before injection, culture samples were centrifuged at 10,000 rpm for 5 min, and the supernatant was filtered through 0.2 µm membrane filters. When necessary, samples were diluted with ultrapure water to bring analyte concentrations within the corresponding calibration range. Dilution factors varied according to the compound, concentration level, and sampling time and were accounted for in the final concentration calculations. Analytical standards of ethanol, glycerol, disodium fumarate, succinic acid, acetic acid, lactic acid, tartaric acid, catechol, and tyrosol were purchased from Merck, with the purities reported in Supplementary Table S1. Compound identification was based on comparison of retention times with injected analytical standards. Quantification was performed using external calibration curves prepared for each target compound. The analytical standards, retention times, calibration ranges, coefficients of determination, limits of detection, and limits of quantification are provided in Supplementary Table S1. Methanol was included in the alcohol inhibitor solution as a minor winery-related alcohol that, although usually present at lower concentrations than ethanol and glycerol, is toxic to biological systems and was therefore considered relevant to the alcohol stress condition. However, due to its low concentration in the experimental medium and the analytical limitations of the HPLC-RID method under the chromatographic conditions used, methanol could not be reliably detected and quantified. Therefore, methanol concentration profiles and consumption rates were not reported, and the alcohol consumption analysis was based on ethanol and glycerol. Condition-specific abiotic blanks containing NBAF medium and the corresponding inhibitor solution, but no inoculum, were analyzed to verify the chromatographic background of each experimental condition and to check for possible interfering peaks at the retention times of the target compounds. In addition, representative samples were spiked with analytical standards covering all target compounds to verify analyte response, retention-time assignment, chromatographic resolution, and possible matrix-related interferences in the experimental matrix. No relevant interfering peaks were observed in the condition-specific blanks at the retention times of the target compounds. In representative spiked samples, the target peaks were confirmed at the expected retention times and remained distinguishable from neighboring peaks, supporting targeted quantification under the chromatographic conditions used.

The specific growth rate (*μ*, h^−1^) was determined from the linearized form of exponential growth, according to Eq. (1), where OD_600(0)_ and OD_600(t)_ represent the blank-corrected optical density at the initial time and at time *t* (h), respectively.

1 $$\mathrm{ln}\left(\frac{{\mathrm{O}\mathrm{D}}_{600(t)}}{{\mathrm{O}\mathrm{D}}_{600(0)}}\right)=\mu \times t$$

The substrate consumption rate (*Q*_CS_, g L^−1^ h^−1^) was calculated using Eq. (2), where *S*_0_ and *S*_t_ (g L^−1^) correspond to the initial and time-dependent substrate concentrations, respectively.

2 $${Q}_{\mathrm{C}\mathrm{S}}=\frac{{S}_{0}-{S}_{\mathrm{t}}}{t}$$

In this study, fumarate consumption was used as a key metabolic readout under anaerobic conditions, and its consumption rate was calculated using Eq. (2). In addition, the consumption rates of ethanol and glycerol (alcohol group), lactic and tartaric acids (acid group), and catechol and tyrosol (phenolic group) were calculated using the same equation. Acetic acid profiles were interpreted as net concentration changes relative to the initial concentration measured in each assay, since acetate was already present in the NBAF medium as sodium acetate, and acetic acid was also added in the carboxylic acid inhibitor condition. Succinic acid profiles were interpreted in the same way because succinate could result from fumarate reduction and, in the carboxylic acid inhibitor condition, was also added as succinic acid. When acetic acid or succinic acid increased during incubation, production rates were calculated using Eq. (2) with reversed numerator terms. Rate calculations were based on the time required to reach the maximum or minimum concentration of each compound, with t denoting the time interval to reach these values.

### Statistical analysis

Growth, consumption, and production rates were statistically analyzed using analysis of variance (ANOVA). All analyses were performed using data from four independent biological replicates per experimental condition, obtained from two independent experimental runs performed in duplicate. Results are presented as mean ± standard deviation. Before ANOVA, Bartlett’s test was applied to verify the homogeneity of variances. Mean comparisons were performed using Fisher’s least significant difference (LSD) test, and differences were considered statistically significant at *p* < 0.05. A two-way ANOVA was conducted to evaluate the effects of inhibitor concentration, microbial consortium (SLL and MSD), and their interaction. For the combined-inhibitor assays, the same statistical approach was applied, considering treatment condition and microbial consortium as factors.

## Results

### Taxonomic composition of the enriched consortia

**Fig. 1:**
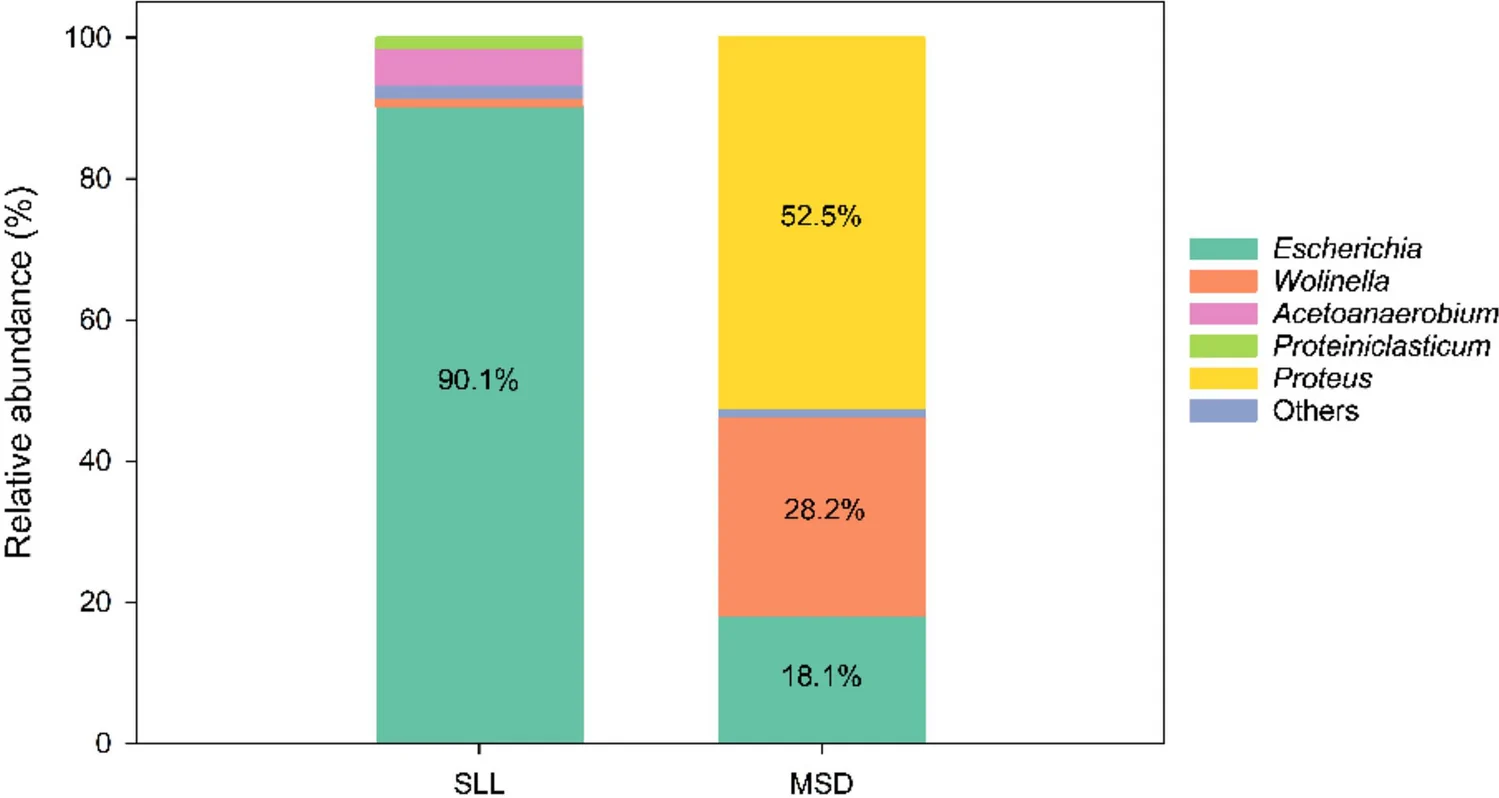
Genus-level taxonomic composition of the enriched SLL and MSD consortia used in the inhibition assays, determined by full-length 16S rRNA gene sequencing. Only genera with a relative abundance ≥ 1% in at least one consortium are shown individually. Genera below this threshold were grouped as “others”. Relative abundances were calculated from classified reads after application of a minimum abundance threshold of 0.01%

Full-length 16S rRNA gene sequencing was used to characterize the bacterial composition of the enriched SLL and MSD consortia used in the inhibition assays. The analysis showed that both inocula consisted of mixed bacterial consortia, with distinct genus-level profiles (Fig. 1 and Supplementary Table S2). No isolation of individual strains was performed.

The SLL consortium was strongly dominated by *Escherichia*, which accounted for 90.1% of the classified reads. Other genera detected above 1% relative abundance included *Acetoanaerobium* (5.3%), *Proteiniclasticum* (1.4%), and *Wolinella* (1.3%). In contrast, the MSD consortium showed a more distributed profile among three dominant genera, with *Proteus* representing 52.5% of the classified reads, followed by *Wolinella* (28.2%) and *Escherichia* (18.1%). Genera below 1% relative abundance were grouped as “others” in Fig. 1 for readability, while the genus-level taxa detected at ≥ 0.1% relative abundance in at least one consortium are provided in Supplementary Table S2. These results show that the enriched SLL and MSD inocula were taxonomically distinct mixed bacterial consortia.

### Consortia development in NBAF medium

**Fig. 2:**
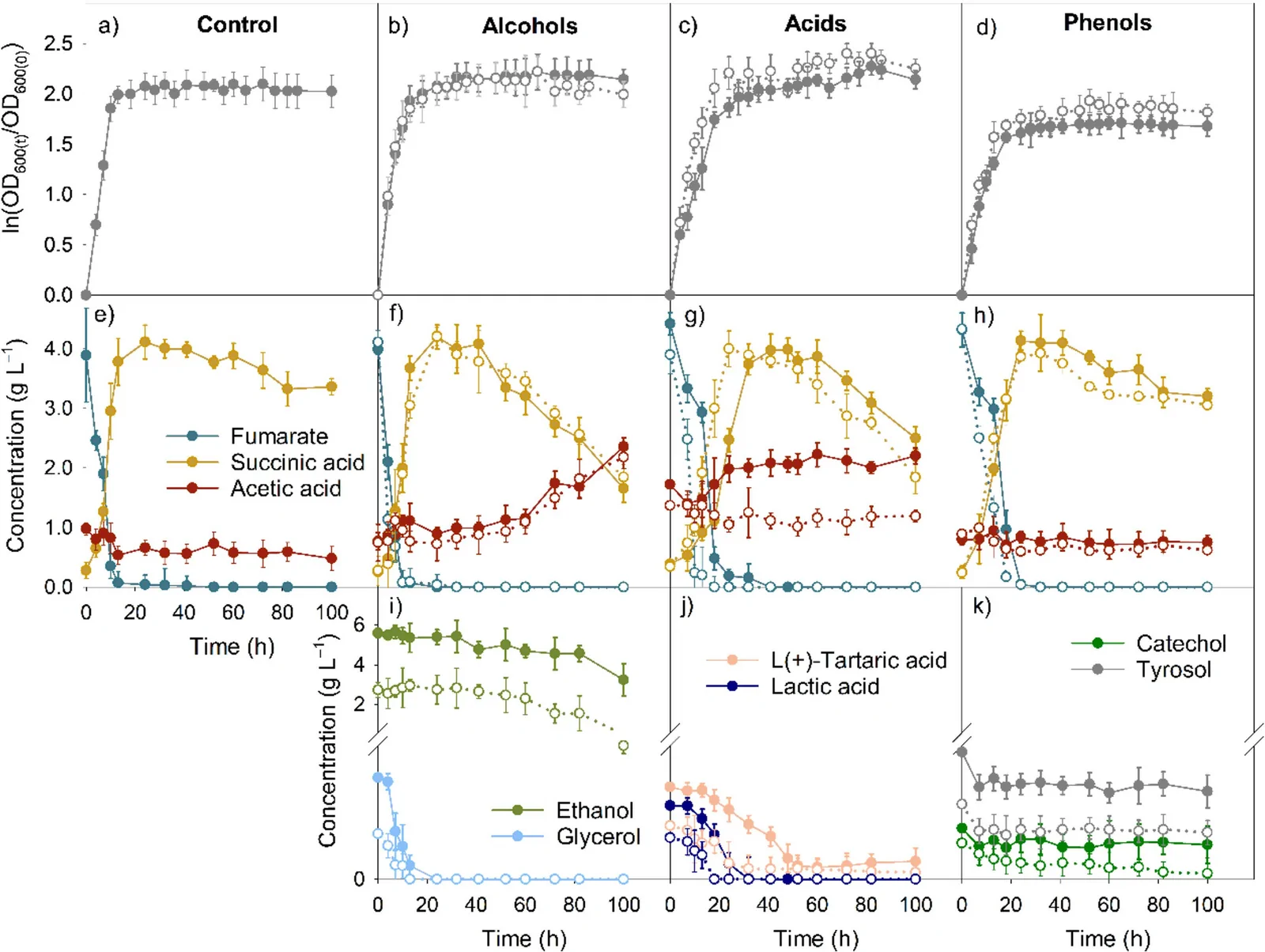
SLL consortium growth curves (**a** to **d**) and fermentation profiles (**e** to **k**) in NBAF medium without inhibitors (**a** and **e**) and in the presence of alcohols (**b**, **f**, and **i**), carboxylic acids (**c**, **g**, and **j**), and phenols (**d**, **h**, and **k**). In the presence of inhibitors, full symbols represent experiments conducted at high inhibitor concentration, and hollow symbols represent experiments conducted at low inhibitor concentration. Data are shown as mean ± standard deviation (*n* = 4 independent biological replicates, obtained from two independent experimental runs performed in duplicate)

**Fig. 3:**
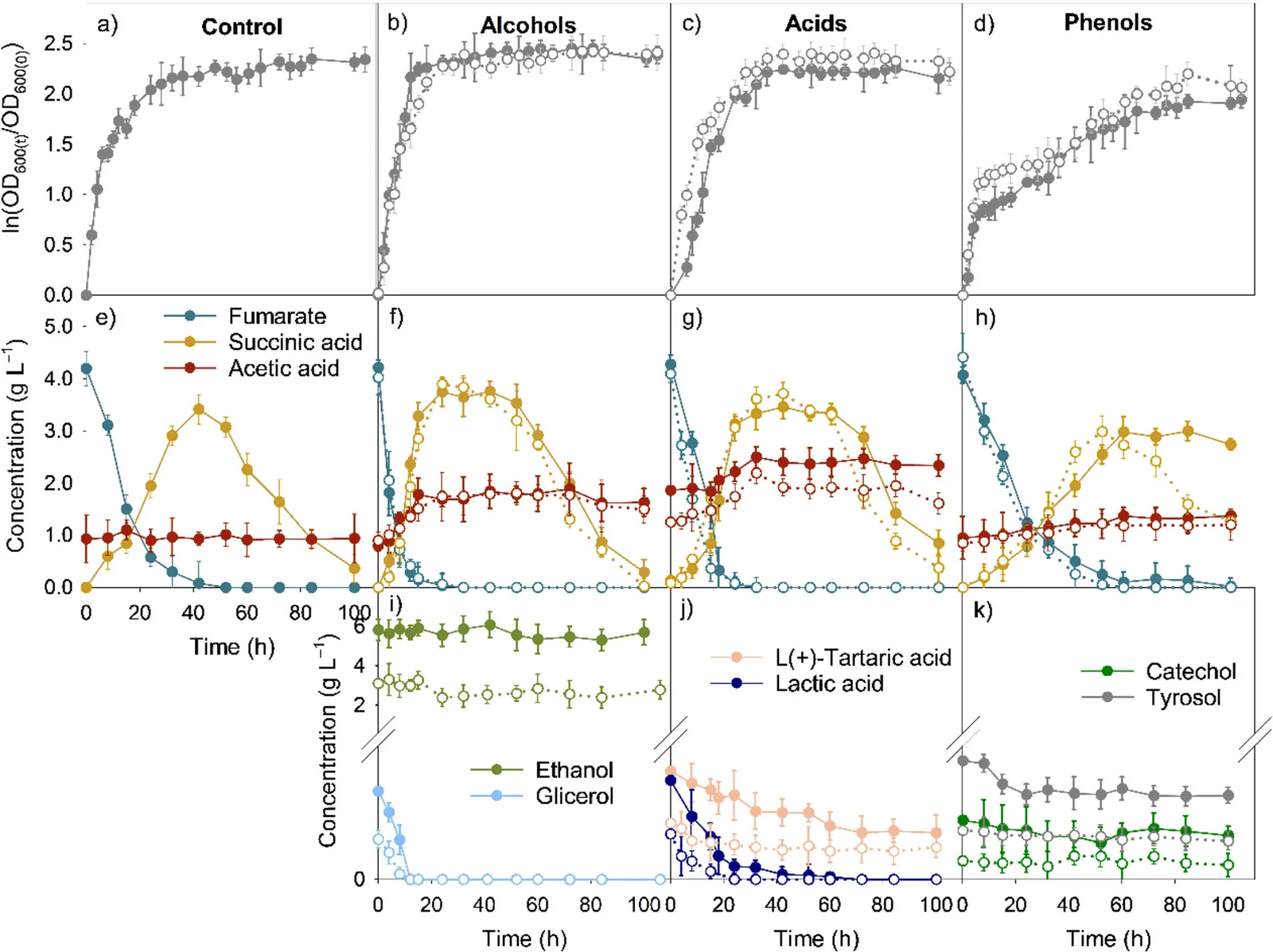
MSD consortium growth curves (**a** to **d**) and fermentation profiles (**e** to **k**) in NBAF medium without inhibitors (a and e) and in the presence of alcohols (**b**, **f**, and **i**), carboxylic acids (**c**, **g**, and **j**), and phenols (**d**, **h**, and **k**). In the presence of inhibitors, full symbols represent experiments conducted at high inhibitor concentration, and hollow symbols represent experiments conducted at low inhibitor concentration. Data are shown as mean ± standard deviation (*n* = 4 independent biological replicates, obtained from two independent experimental runs performed in duplicate)

Both consortia exhibited similar specific growth rates under control conditions (0.24 h^−1^ for SLL and 0.26 h^−1^ for MSD; Table 2), with no statistically significant difference between them. However, differences in growth dynamics were observed. The SLL consortium showed a rapid exponential phase within the first 10–12 h, followed by an early transition to the stationary phase (Fig. 2a). In contrast, MSD exhibited a longer lag phase and reached the stationary phase after approximately 40 h (Fig. 3a).

**Table 2:** Growth and metabolic rates of SLL and MSD consortia in the presence of alcohols

Consortium	Condition	Specific growth rate (h^−1^)	Fumarate consumption rate (g L^−1^ h^−1^)	Ethanol consumption rate (g L^−1^ h^−1^)	Glycerol consumption rate (g L^−1^ h^−1^)	Acetic acid production rate (g L^−1^ h^−1^)	Succinic acid production rate (g L^−1^ h^−1^)
SLL	Control	0.24 ± 0.06^ab^	0.38 ± 0.07^a^	–	–	−0.004 ± 0.001^d^	0.19 ± 0.01^a^
SLL	LC	0.20 ± 0.08^b^	0.34 ± 0.06^a^	0.023 ± 0.005^a^	0.016 ± 0.005^b^	0.015 ± 0.008^a^	0.18 ± 0.01^a^
SLL	HC	0.22 ± 0.04^ab^	0.36 ± 0.03^a^	0.020 ± 0.007^a^	0.018 ± 0.007^b^	0.019 ± 0.005^a^	0.17 ± 0.06^a^
MSD	Control	0.26 ± 0.03^a^	0.16 ± 0.01^b^	–	–	0.00 ± 0.01^c^	0.09 ± 0.05^b^
MSD	LC	0.24 ± 0.05^ab^	0.17 ± 0.01^b^	0.010 ± 0.005^b^	0.017 ± 0.004^b^	0.010 ± 0.008^b^	0.18 ± 0.07^a^
MSD	HC	0.22 ± 0.05^ab^	0.18 ± 0.02^b^	0.008 ± 0.004^b^	0.024 ± 0.008^a^	0.013 ± 0.005^b^	0.17 ± 0.08^a^
ANOVA	Consortium	**	***	***	ns	***	***
ANOVA	inhibitor	ns	ns	***	***	***	***
ANOVA	Consortium × inhibitor	ns	ns	***	ns	***	***

Values are presented as mean ± standard deviation (*n* = 4 independent biological replicates, obtained from two independent experimental runs performed in duplicate). Different letters within each variable indicate significant differences according to Fisher’s LSD test following ANOVA (*p* < 0.05). En dashes “–” indicate not applicable

*ns* not significant, *LC* low concentration, *HC* high concentration

^*^Significance at *p* < 0.05 (ANOVA results)

^**^Significance at *p* < 0.01 (ANOVA results)

^**^^*^Significance at *p* < 0.001 (ANOVA results)

Fumarate consumption also differed between consortia. In SLL, fumarate was depleted within approximately 13 h, whereas in MSD, depletion required more than twice that time (Figs. 2e and 3e). Correspondingly, fumarate consumption rates were higher in SLL (0.38 g L^−1^ h^−1^) than in MSD (0.16 g L^−1^ h^−1^; Table 2). In both consortia, fumarate reduction was associated with succinic acid production, exhibiting temporal trends similar to those of substrate depletion.

Differences were also observed in succinate profiles. In SLL, succinate accumulated to higher concentrations and was only partially consumed after reaching its maximum. In contrast, in MSD, succinate was almost completely depleted following its peak concentration (Figs. 2e and 3e). Acetic acid profiles further differed, with partial consumption observed in SLL, whereas MSD showed relatively stable acetate concentrations throughout the experiment.

### Effect of alcohol inhibitors

Alcohol exerted a limited effect on microbial growth. Both consortia showed only slight reductions in growth rates compared to control conditions, with no significant differences between low and high concentrations (Table 2; Figs. 2b and 3b). Growth curves remained similar to those under control conditions, with no major changes in lag or stationary phase durations.

Fumarate consumption remained unaffected by alcohol addition, with SLL maintaining higher rates (0.34–0.36 g L^−1^ h^−1^) than MSD (0.17–0.18 g L^−1^ h^−1^; Table 2). Substrate depletion profiles were comparable to those observed under control conditions for both consortia. However, differences were observed in metabolite production. In SLL, succinate production remained similar to control values, whereas in MSD, it increased significantly under alcohol exposure. Acetic acid profiles showed transient production in SLL, particularly following glycerol consumption, whereas MSD exhibited earlier but lower acetate production (Figs. 2f and i and 3f and i).

Ethanol consumption rates were higher in SLL (0.023 g L^−1^ h^−1^) than in MSD (0.010 g L^−1^ h^−1^), while glycerol consumption showed greater variability between conditions, reaching its highest values under high-inhibitor concentrations (Table 2).

### Effect of carboxylic acid inhibitors

Carboxylic acids significantly reduced growth rates in both consortia (*p* < 0.01), decreasing from an average of 0.25 h^−1^ under control conditions to 0.14 h^−1^ in the presence of inhibitors (Table 3). This reduction was observed under both low- and high-concentration conditions.

**Table 3:** Growth and metabolic rates of SLL and MSD consortia in the presence of carboxylic acids

Consortium	Condition	Specific growth rate (h^−1^)	Fumarate consumption rate (g L^−1^ h^−1^)	Lactic acid consumption rate (g L^−1^ h^−1^)	Tartaric acid consumption rate (g L^−1^ h^−1^)	Acetic acid production rate (g L^−1^ h^−1^)	Succinic acid production rate (g L^−1^ h^−1^)
SLL	Control	0.24 ± 0.06^ab^	0.38 ± 0.07^a^	–	–	−0.004 ± 0.001^d^	0.19 ± 0.01^a^
SLL	LC	0.16 ± 0.05^b^	0.24 ± 0.07^a^	0.015 ± 0.004^a^	0.013 ± 0.005^a^	−0.002 ± 0.001^ cd^	0.18 ± 0.03^a^
SLL	HC	0.13 ± 0.02^b^	0.15 ± 0.04^b^	0.014 ± 0.006^a^	0.013 ± 0.003^a^	0.006 ± 0.001^ab^	0.11 ± 0.04^c^
MSD	Control	0.26 ± 0.03^a^	0.16 ± 0.01^b^	–	–	0.00 ± 0.01^c^	0.09 ± 0.05^b^
MSD	LC	0.15 ± 0.04^b^	0.15 ± 0.06^b^	0.013 ± 0.003^b^	0.004 ± 0.001^c^	0.004 ± 0.002^b^	0.14 ± 0.04^b^
MSD	HC	0.13 ± 0.01^b^	0.17 ± 0.02^b^	0.012 ± 0.004^b^	0.007 ± 0.002^b^	0.007 ± 0.002^a^	0.12 ± 0.03^bc^
ANOVA	Consortium	ns	***	***	***	***	***
ANOVA	inhibitor	**	*	***	***	***	**
ANOVA	Consortium × inhibitor	ns	*	**	***	*	***

Values are presented as mean ± standard deviation (*n* = 4 independent biological replicates, obtained from two independent experimental runs performed in duplicate). Different letters within each variable indicate significant differences according to Fisher’s LSD test following ANOVA (*p* < 0.05). En dashes “–” indicate not applicable

*ns* not significant, *LC* low concentration, *HC* high concentration

^*^Significance at *p* < 0.05 (ANOVA results)

^**^Significance at *p* < 0.01 (ANOVA results)

^**^^*^Significance at *p* < 0.001 (ANOVA results)

Fumarate consumption profiles remained relatively stable in MSD but decreased significantly in SLL at high inhibitor concentration (0.15 vs. 0.38 g L^−1^ h^−1^; Table 3). Despite these differences in consumption rates, fumarate depletion was still observed in both consortia under carboxylic acid exposure.

Marked differences were observed in succinic acid production. In SLL, no significant variation was observed at low concentration, but a decrease was observed at high concentration. In contrast, MSD showed higher succinate production values in the presence of carboxylic acids, although the differences among MSD conditions were less pronounced than those observed for SLL. Temporal profiles showed delayed succinate accumulation in SLL at higher concentrations, whereas MSD showed less pronounced changes in succinate profiles between control and acid-supplemented conditions.

Acetic acid profiles also differed between consortia. In SLL, acetate shifted from consumption under control conditions to production at high inhibitor concentration. In MSD, acetate production increased with inhibitor concentration. Lactic acid was readily consumed in both consortia, whereas tartaric acid consumption was slower and lower in MSD.

### Effect of phenolic inhibitors

Phenolic compounds exerted a strong inhibitory effect on growth, particularly at the high-concentration level. Growth rates decreased significantly in both consortia (*p* < 0.01), with SLL decreasing from 0.24 h^−1^ under control conditions to 0.11 h^−1^ at high phenolic concentration, and MSD decreasing from 0.26 to 0.10 h^−1^ (Table 4; Figs. 2d and 3d). Growth profiles showed a delayed response in MSD, characterized by an extended lag phase.

**Table 4:** Growth and metabolic rates of SLL and MSD consortia in the presence of phenolic compounds

Consortium	Condition	Specific growth rate (h^−1^)	Fumarate consumption rate (g L^−1^ h^−1^)	Catechol consumption rate (g L^−1^ h^−1^)	Tyrosol consumption rate (g L^−1^ h^−1^)	Acetic acid production rate (g L^−1^ h^−1^)	Succinic acid production rate (g L^−1^ h^−1^)
SLL	Control	0.24 ± 0.06^ab^	0.38 ± 0.07^a^	–	–	−0.004 ± 0.001^d^	0.19 ± 0.01^a^
SLL	LC	0.13 ± 0.03^b^	0.22 ± 0.06^b^	0.014 ± 0.005^a^	0.0003 ± 0.0002^b^	−0.001 ± 0.001^d^	0.19 ± 0.05^a^
SLL	HC	0.11 ± 0.05^b^	0.16 ± 0.04^b^	0.011 ± 0.003^b^	0.002 ± 0.001^a^	0.00 ± 0.01^ cd^	0.18 ± 0.03^a^
MSD	Control	0.26 ± 0.03^a^	0.16 ± 0.01^b^	–	–	0.00 ± 0.01^c^	0.09 ± 0.05^b^
MSD	LC	0.21 ± 0.01^a^	0.17 ± 0.05^b^	0.002 ± 0.001^c^	0.002 ± 0.001^c^	0.004 ± 0.002^b^	0.07 ± 0.02^c^
MSD	HC	0.10 ± 0.03^b^	0.15 ± 0.04^b^	0.003 ± 0.002^c^	0.003 ± 0.001^a^	0.006 ± 0.004^a^	0.05 ± 0.02^c^
ANOVA	Consortium	ns	*	***	*	***	***
ANOVA	Inhibitor	**	ns	***	***	***	*
ANOVA	Consortium × inhibitor	ns	ns	***	***	*	ns

Values are presented as mean ± standard deviation (*n* = 4 independent biological replicates, obtained from two independent experimental runs performed in duplicate). Different letters within each variable indicate significant differences according to Fisher’s LSD test following ANOVA (*p* < 0.05). En dashes “–” indicate not applicable

*ns* not significant, *LC* low concentration, *HC* high concentration

^*^Significance at *p* < 0.05 (ANOVA results)

^**^Significance at *p* < 0.01 (ANOVA results)

^**^^*^Significance at *p* < 0.001 (ANOVA results)

Fumarate consumption decreased in SLL as inhibitor concentration increased but remained relatively stable in MSD (Table 4). Succinic acid production decreased in MSD compared to control conditions but remained similar in SLL. Acetic acid profiles showed distinct patterns: SLL shifted toward a near-zero balance at high concentrations, whereas MSD exhibited increased acetate production. Catechol and tyrosol showed only a small concentration decrease in both consortia, consistent with low apparent consumption rates (Table 4; Figs. 2k and 3k).

### Combined inhibitory conditions

The combined-inhibitor assays evaluated the response of each consortium to simultaneous exposure to alcohols, carboxylic acids, and phenolic compounds at the high-concentration level. Combined inhibitors significantly reduced growth rates in both consortia, decreasing to approximately 0.15 h^−1^ in SLL and 0.13 h^−1^ in MSD (Table 5). Growth curves showed extended lag phases and reduced biomass accumulation compared to control conditions.

**Table 5:** Growth and metabolic rates of SLL and MSD consortia under combined inhibitory conditions

Consortium	Condition	Specific growth rate (h^−1^)	Fumarate consumption rate (g L^−1^ h^−1^)	Ethanol consumption rate (g L^−1^ h^−1^)	Glycerol consumption rate (g L^−1^ h^−1^)	Lactic acid consumption rate (g L^−1^ h^−1^)	Tartaric acid consumption rate (g L^−1^ h^−1^)	Catechol consumption rate (g L^−1^ h^−1^)	Tyrosol consumption rate (g L^−1^ h^−1^)	Acetic acid production rate (g L^−1^ h^−1^)	Succinic acid production rate (g L^−1^ h^−1^)
SLL	Control	0.36 ± 0.04^a^	0.19 ± 0.07^a^	–	–	–	–	–	–	−0.002 ± 0.001^d^	0.23 ± 0.06^c^
SLL	HC	0.15 ± 0.06^c^	0.17 ± 0.08^a^	0.02 ± 0.01^a^	0.02 ± 0.01^a^	0.006 ± 0.002^a^	0.004 ± 0.002^a^	0.0010 ± 0.0005	0.0006 ± 0.0001	0.008 ± 0.001^a^	0.35 ± 0.07^b^
MSD	Control	0.32 ± 0.07^b^	0.12 ± 0.05^b^	–	–	–	–	–	–	0.002 ± 0.001^c^	0.07 ± 0.02^d^
MSD	HC	0.13 ± 0.05^c^	0.19 ± 0.08^a^	−0.003 ± 0.001^b^	0.009 ± 0.003^b^	0.002 ± 0.001^b^	0.002 ± 0.001^b^	0.00010 ± 0.00007	−0.0002 ± 0.0001	0.003 ± 0.002^b^	0.41 ± 0.06^a^
ANOVA	Consortium	ns	***	**	***	***	***	ns	ns	*	**
ANOVA	Inhibitor	***	***	*	***	***	***	ns	ns	***	***
ANOVA	Consortium × INHIBITOR	*	***	**	***	***	***	ns	ns	***	***

Values are presented as mean ± standard deviation (*n* = 4 independent biological replicates, obtained from two independent experimental runs performed in duplicate). Different letters within each variable indicate significant differences according to Fisher’s LSD test following ANOVA (*p* < 0.05). En dashes “–” indicate not applicable

*ns* not significant, *LC* low concentration, *HC* high concentration

^*^Significance at *p* < 0.05 (ANOVA results)

^**^Significance at *p* < 0.01 (ANOVA results)

^**^^*^Significance at *p* < 0.001 (ANOVA results)

**Fig. 4:**
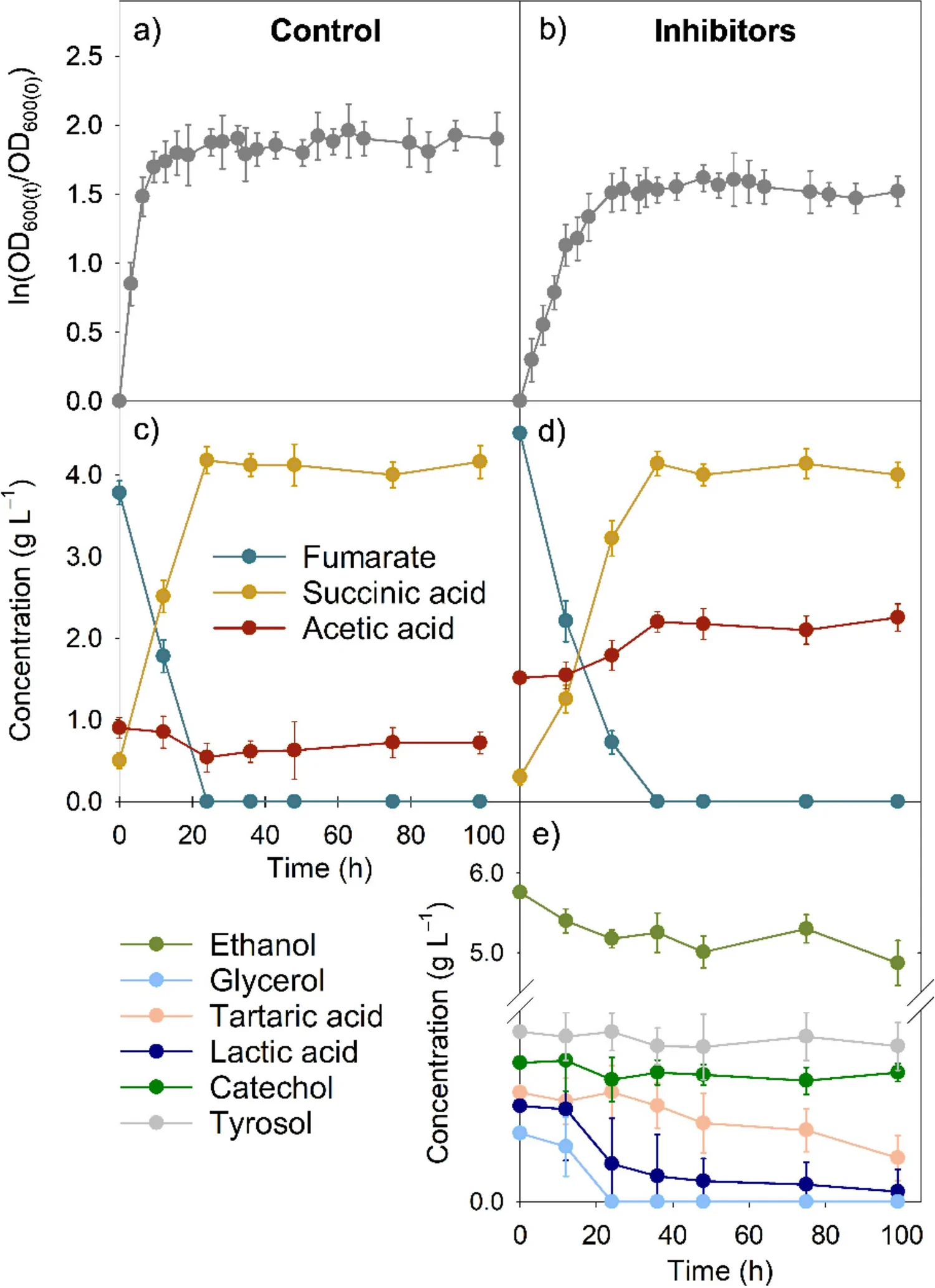
SLL consortium growth curves (**a** and **b**) and fermentation profiles (**c** to **e**) in NBAF medium without inhibitors (**a** and **c**) and in the presence of combined inhibitors at the high-concentration level (**b**, **d**, and **e**). Data are shown as mean ± standard deviation (*n* = 4 independent biological replicates, obtained from two independent experimental runs performed in duplicate)

**Fig. 5:**
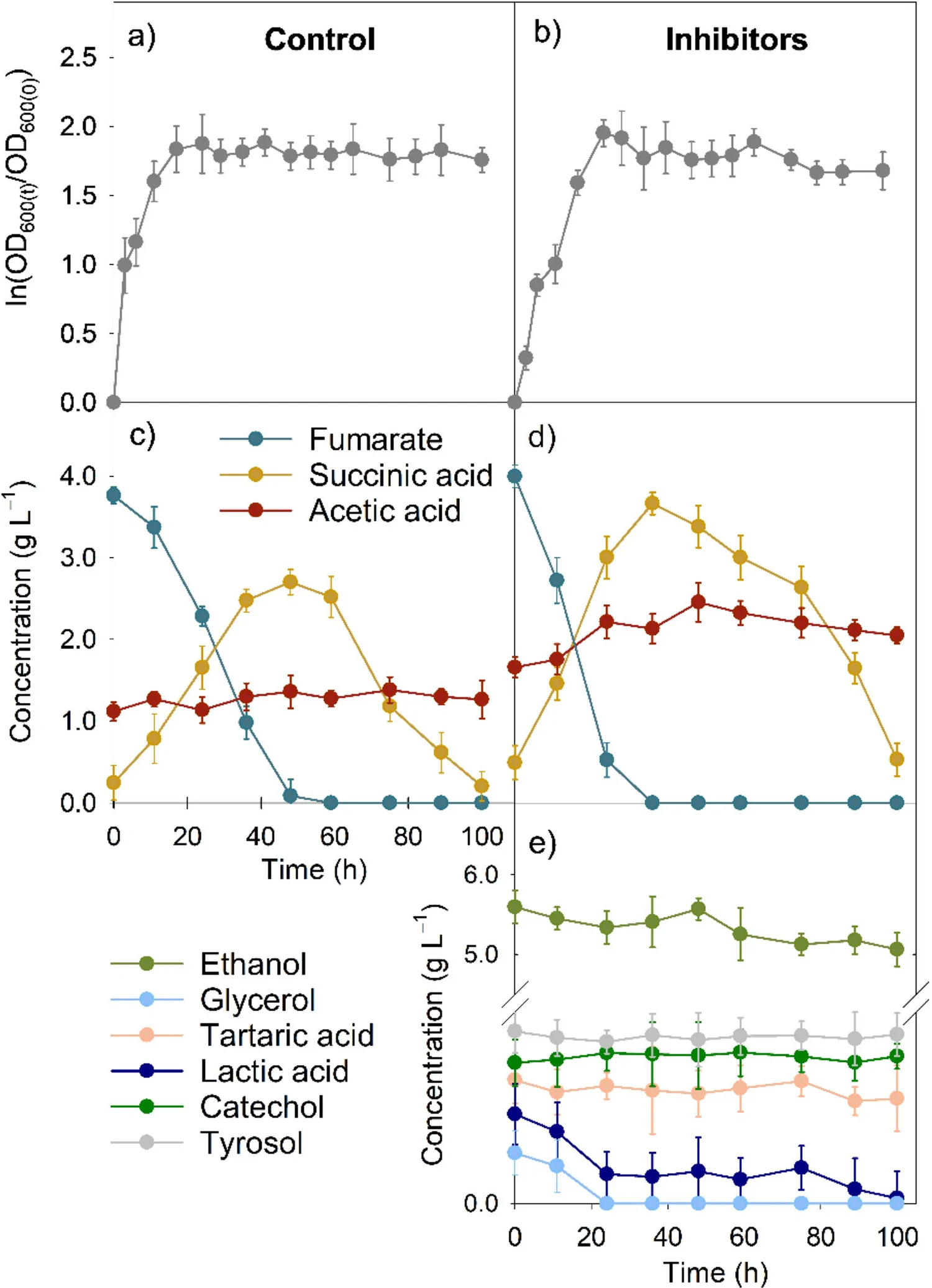
MSD consortium growth curves (**a** and **b**) and fermentation profiles (**c** to **e**) in NBAF medium without inhibitors (**a** and **c**) and in the presence of combined inhibitors at the high-concentration level (**b**, **d**, and **e**). Data are shown as mean ± standard deviation (*n* = 4 independent biological replicates, obtained from two independent experimental runs performed in duplicate)

Despite this reduction in growth, fumarate consumption remained active. In SLL, consumption rates remained relatively stable, although total depletion time increased. In MSD, fumarate consumption rates increased compared to control conditions, with faster substrate depletion observed (Figs. 4d and 5d).

Succinic acid production increased in both consortia under combined inhibitory conditions, particularly in MSD (up to 0.41 g L^−1^ h^−1^; Table 5). Acetic acid profiles also changed, with SLL shifting from net consumption to net production, whereas MSD showed lower production compared to isolated inhibitor conditions.

Consumption of the added inhibitor substrates was generally lower under combined conditions, although the response depended on the compound and consortium. Lactic and tartaric acids showed lower consumption rates under the combined condition, particularly in MSD. Ethanol and glycerol were consumed in SLL, whereas MSD showed little or no apparent ethanol consumption and lower glycerol consumption. Catechol and tyrosol showed only minor concentration changes, with no significant enhancement in their apparent consumption under combined conditions.

## Discussion

The results demonstrate that microbial consortia with similar growth rates can exhibit markedly different metabolic behaviors, highlighting the importance of microbial origin in determining carbon flux distribution under anaerobic conditions. Under control conditions and in the presence of alcohols, the SLL consortium showed faster fumarate consumption and higher substrate turnover than MSD. However, this difference was not maintained under combined stress conditions. In MSD, fumarate consumption remained comparatively stable in the presence of carboxylic acids and phenolic compounds and increased under combined inhibitory conditions, indicating a stress-dependent metabolic response. Thus, SLL appears to be characterized by faster baseline turnover, whereas MSD shows a more stress-resilient fumarate-consuming phenotype. These differences may be related, at least in part, to the contrasting environmental origin and taxonomic composition of each enriched consortium. The SLL consortium was derived from mature landfill leachate, an environment typically associated with fermentative, acetogenic, and methanogenic microorganisms (Adhikari and Khanal 2015; Ishaq et al. 2024), which is consistent with its rapid substrate turnover under control and alcohol-supplemented conditions. In contrast, the MSD consortium originated from an acidic, metal-rich mining environment, where microbial communities are commonly exposed to chemically harsh conditions (Mendonça et al. 2003). Similar communities have been described in acid mine drainage systems such as the Tinto River (Fernández-Remolar et al. 2003), where syntrophic interactions play a key role in sustaining metabolic activity under energy-limited conditions. Nevertheless, because both consortia were enriched for 3 months in the same acetate–fumarate medium, the observed metabolic profiles should not be attributed solely to the original inoculum source. They likely reflect the combined influence of the initial microbial source, the enrichment process, and the inhibitory conditions applied in the assays.

From a metabolic perspective, the fumarate, succinate, and acetate profiles helped distinguish how the two consortia responded to the defined anaerobic medium and to inhibitor exposure. In assays without inhibitors and in alcohol-supplemented assays, SLL consumed fumarate faster than MSD and accumulated higher succinate concentrations, indicating faster fumarate-to-succinate conversion. This response was more affected by carboxylic acid and phenolic stress in SLL, whereas MSD maintained fumarate consumption more consistently across these inhibitor conditions and showed increased fumarate consumption under combined inhibitory conditions.

Succinate profiles provided an additional distinction between the consortia. In SLL, succinate accumulated to higher concentrations and was only partially consumed after reaching its maximum, whereas in MSD, succinate decreased more markedly after accumulation. This indicates that succinate behaved mainly as an accumulated product in SLL, while in MSD it may also have been further converted or incorporated into downstream metabolism after its formation. In anaerobic systems, the further conversion of organic acid intermediates can depend on thermodynamically constrained and cooperative processes, including syntrophic interactions and shifts in acetate metabolism (Pan et al. 2021; Jin and Lu 2023). Such interactions have also been associated with volatile fatty acid turnover and maintenance of metabolic activity under inhibitory conditions (Nikitina et al. 2023). However, these mechanisms were not directly demonstrated in the present study and should therefore be interpreted only as possible explanations for the observed succinate profiles.

Acetate profiles also differed between consortia, but they require cautious interpretation because acetate was already present in the NBAF medium and was also added in the carboxylic acid condition. Therefore, changes in acetate concentration were interpreted as net balances between production and consumption rather than as the output of a single pathway. These metabolic readouts are relevant because they show whether enriched consortia can maintain organic acid turnover and intermediate conversion in the presence of inhibitory compounds.

Alcohols had a limited impact on microbial growth, indicating that these compounds were less inhibitory than carboxylic acids and phenolic compounds under the tested conditions. This is consistent with the fact that simple alcohols can be used as carbon and electron sources by facultative anaerobes, including *Escherichia coli*, and can be connected to central anaerobic metabolic routes (Schubert and Unden 2023). In contrast, carboxylic acids significantly affected growth, likely due to weak-acid stress mechanisms. At near-neutral pH, carboxylic acids are predominantly in their dissociated form. Still, at higher concentrations, the undissociated fraction can diffuse across cell membranes, leading to intracellular acidification and increased energetic demand for pH homeostasis (Gaspar et al. 2025). These results are consistent with the known anaerobic metabolism of organic acids, in which simpler compounds such as lactate can be readily metabolized, whereas more complex acids such as tartaric acid require additional enzymatic steps (Schubert and Unden 2022; Parshina et al. 2023). The importance of acid tolerance has also been demonstrated in alternative biological systems, such as acid-tolerant microalgae (Praveen et al. 2025).

Phenolic compounds exerted a strong inhibitory effect, particularly at the high-concentration level, consistent with their well-documented toxicity toward microbial cells. Phenols can disrupt membrane integrity, interfere with enzymatic activity, and inhibit key metabolic pathways, particularly those associated with anaerobic energy metabolism (Wang et al. 1989, 1991; Baral and Shah 2014). The small concentration decreases observed for catechol and tyrosol indicate that these compounds were only weakly affected under the tested anaerobic conditions. These decreases may reflect limited biological conversion, adsorption to biomass, abiotic transformation, analytical uncertainty, or partial conversion into unidentified products. Therefore, catechol and tyrosol were interpreted as poorly transformed target compounds under the conditions tested, in line with the generally recalcitrant behavior of phenolic compounds reported in anaerobic biodegradation studies (Biswal et al. 2023). Efficient transformation of phenolic compounds under anaerobic conditions often requires specialized microbial populations (Levén et al. 2012), which may have been absent, present at low relative abundance, or not metabolically active under the conditions tested.

Under combined inhibitory conditions, a clear decoupling between growth and metabolic activity was observed. Despite reduced biomass formation, fumarate consumption and metabolite production remained active, suggesting that metabolic activity related to electron-acceptor use and intermediate formation was maintained under combined inhibitory conditions. This was particularly evident in MSD, where fumarate consumption increased relative to the corresponding control condition, together with enhanced succinate production. This suggests that, although MSD was slower than SLL under non-stressed or mildly stressed conditions, exposure to the combined inhibitor mixture was associated with a stronger apparent contribution of fumarate reduction as an electron-accepting route. The greater availability of organic substrates may have contributed to the observed increase in metabolite production. However, because the combined condition included alcohols, carboxylic acids, and phenolic compounds simultaneously, the relative contribution of each inhibitor group, pH-related effects, and possible interactions among compounds cannot be distinguished from the present experimental design. Similar redistribution of metabolic fluxes under anaerobic conditions has been described in metabolic modeling studies of microbial consortia (Lee et al. 2024).

From a microbiological perspective, the observed metabolic patterns can be discussed in light of the distinct genus-level composition of the enriched consortia, although taxonomic data alone do not demonstrate function (Douglas et al. 2020). The SLL consortium was strongly dominated by *Escherichia*, with lower contributions of *Acetoanaerobium*, *Proteiniclasticum*, and *Wolinella* (Fig. 1 and Supplementary Table S2). This composition is compatible with the rapid fumarate consumption and succinate accumulation observed under control and alcohol-supplemented conditions, since facultative anaerobes such as *Escherichia* can use fumarate as an electron acceptor under anaerobic conditions (Iverson et al. 1999). The presence of anaerobic and fermentative genera at lower abundance, such as *Acetoanaerobium* and *Proteiniclasticum*, may also have contributed to the overall fermentation profiles, although their specific activity was not directly assessed (Sleat et al. 1985; Zhang et al. 2010).

The MSD consortium showed a more distributed taxonomic profile, dominated by *Proteus*, *Wolinella*, and *Escherichia* (Fig. 1 and Supplementary Table S2). This broader distribution may be consistent with the more conserved fumarate-consuming response observed under carboxylic acid and phenolic stress and with the increased fumarate consumption under combined inhibitory conditions, since fumarate respiration has been described in *Wolinella succinogenes* and fumarate reductase activity has also been reported in *Proteus mirabilis* under anaerobic conditions (Kröger et al. 2002; Van der Beek et al. 1976). The maintenance of fumarate consumption and intermediate metabolite turnover under inhibitory conditions may involve cooperative interactions, including syntrophic processes, which are known to depend on metabolic networks that regulate performance in complex microbial consortia (Jin and Lu 2023; Toczyłowska-Mamińska et al. 2020). These interactions can contribute to volatile fatty acid turnover and help sustain metabolic activity under inhibitory conditions (Nikitina et al. 2023). However, the specific microbial groups and interaction mechanisms involved were not directly demonstrated in the present study. Functional roles cannot be assigned directly from 16S rRNA gene data alone, and the links between community composition, enrichment history, and metabolic performance should therefore be interpreted as putative and require confirmation by functional approaches, such as metagenomics, metatranscriptomics, enzyme activity assays, or targeted activity measurements.

Although the functional mechanisms remain unresolved, the observed sensitivity to inhibitory compounds is consistent with previous studies showing that reducing inhibitor loads can improve biological performance. For instance, Mahmoud et al. (2016) reported enhanced conversion of organic matter following Fenton pretreatment, while Alves-Ferreira et al. (2022) demonstrated improved productivity after phenolic removal. Integrated treatment approaches have also been shown to enhance overall system performance in winery wastewater treatment (Scarponi et al. 2024).

The tolerance patterns observed in this study are particularly relevant in the context of anaerobic treatment of winery and other agro-industrial wastewaters. Although many aerobic microorganisms can tolerate and degrade alcohols, carboxylic acids, and phenolic compounds, the advantage of anaerobic consortia is mainly process-related rather than based on higher intrinsic tolerance. Aerobic treatment relies on continuous aeration, which increases energy demand and may become less efficient when inhibitory compounds reduce oxygen uptake, biomass activity, or degradation rates. In contrast, anaerobic systems avoid the need for aeration and can redirect part of the organic load toward energy-rich products, particularly methane or, in bioelectrochemical configurations, hydrogen (Logan et al. 2008, 2023). Under anaerobic conditions, the conversion of organic matter depends on the coordinated activity of fermentative, syntrophic, acetogenic, and methanogenic populations. When these trophic interactions are balanced, intermediate metabolites such as volatile fatty acids can be further converted to methane and CO_2_, supporting extensive biodegradation of complex organic matter. The different responses of the SLL and MSD consortia, therefore, indicate that tolerance should not be evaluated only from growth inhibition, but also from the ability to maintain substrate turnover and metabolite conversion under oxygen-free conditions.

The present results should be interpreted as evidence of differential phenotypic and metabolic responses of two enriched consortia under inhibitory conditions, rather than as direct demonstration of long-term microbial adaptation or as evidence that the original inoculum source alone determined the final metabolic behavior. Because both consortia were enriched for 3 months in the same acetate–fumarate medium, the observed responses likely reflect the combined influence of the original microbial source, the enrichment process, and the imposed inhibitory conditions. Confirmation of adaptation in the strict sense would require serial exposure experiments and functional validation of the metabolic mechanisms involved.

The obtained results highlight that microbial source, enrichment history, and inhibitory conditions jointly shaped the resilience and differential metabolic responses observed under chemically complex conditions. These findings may help guide the selection of microbial consortia for future anaerobic treatment studies and, after appropriate validation, for bioelectrochemical systems aimed at agro-industrial wastewater valorization (Bayon-Vicente et al. 2025). The use of microbial consortia has been widely reported as an effective strategy for resource recovery, including electricity and biomass generation (Nayak et al. 2024).

## Conclusion

This study shows that the resilience of anaerobic microbial consortia to winery wastewater-associated inhibitors is not determined by growth alone, but by their capacity to maintain substrate turnover and metabolite conversion under stress. Although both consortia showed comparable growth rates in the absence of inhibitors, they differed markedly in growth dynamics, fumarate consumption, succinate and acetate profiles, and inhibitor consumption patterns. The SLL consortium was characterized by rapid fumarate consumption and succinate accumulation under control and alcohol-supplemented conditions, indicating faster baseline substrate turnover. This response was consistent with its strong dominance by *Escherichia*, together with lower-abundance anaerobic or fermentative genera. In contrast, the MSD consortium showed a more distributed taxonomic profile, dominated by *Proteus*, *Wolinella*, and *Escherichia*, and maintained fumarate consumption more consistently under carboxylic acid and phenolic stress. Under combined inhibitory conditions, MSD also showed increased fumarate consumption and enhanced succinate production, suggesting a distinct stress-associated metabolic response.

Among the tested inhibitor groups, alcohols had limited effects on growth and fumarate consumption, whereas carboxylic acids significantly reduced growth and altered metabolite profiles. Phenolic compounds exerted a strong inhibitory effect, particularly at the high-concentration level, and catechol and tyrosol showed only minor concentration changes, indicating low apparent consumption under the anaerobic conditions tested. The combined-inhibitor assays further showed that growth inhibition did not necessarily prevent fumarate consumption or metabolite formation, highlighting the importance of evaluating both biomass formation and metabolic activity when assessing consortium tolerance. These results indicate that microbial source, enrichment history, taxonomic composition, and inhibitory conditions jointly shaped the resilience and metabolic performance of the enriched consortia. The observed links between community composition and metabolic behavior remain putative and should be confirmed through serial exposure experiments and functional analyses. The present study should be viewed as a controlled screening step designed to compare microbial responses under defined acetate–fumarate conditions. Further testing in real winery wastewater and more complex treatment systems will be needed before selecting the most suitable consortia for practical applications. Within this scope, the results support the use of non-conventional anaerobic consortia as candidates for future wastewater treatment studies involving chemically complex agro-industrial effluents.

## Supplementary Information

Below is the link to the electronic supplementary material.

ESM 1 (DOCX 56.2 KB)

## Data Availability

Data and materials will be made available on request.
