# Supplementary material for: Metabolic responses of microbial consortia to winery wastewater-associated inhibitors
Source: Appl Microbiol Biotechnol. 2026 Aug 3;110:322. doi: 10.1007/s00253-026-13983-y (PMC13719931; doi:10.1007/s00253-026-13983-y)
Supplement: Supplementary file 1 [file 253_2026_13983_MOESM1_ESM.docx]

**Metabolic responses of microbial consortia to winery wastewater-associated inhibitors**

Ana Baía^1,2^, Alonso I. Arroyo-Escoto^3,4,5^, Bilel Abdelkarim^1^, Nuno Ramos^2^, César Afonso^1,2^, Gaetano R. Pesce^6^, Maria C. Fernandes^3,4,*^, Annabel Fernandes^1,2,*^

**Supplementary Information**

**Table S1.** Analytical standards and HPLC calibration parameters used for compound identification and quantification.

| Compound | Supplier | Purity  (%) | Retention time  (min) | Calibration range  (mg L^−1^) | R^2^ | LOD  (mg L^−1^) | LOQ  (mg L^−1^) |
| --- | --- | --- | --- | --- | --- | --- | --- |
| Ethanol | Merck | ≥99.5 | 22.486 | 5−250 | 0.9988 | 1.39 | 4.57 |
| Glycerol | Merck | ≥99.5 | 13.579 | 2−100 | 0.9987 | 0.43 | 1.42 |
| Disodium fumarate | Merck | ≥99 | 14.137 | 5−250 | 0.9993 | 1.36 | 4.45 |
| Succinic acid | Merck | ≥99.0 | 11.774 | 5−250 | 0.9994 | 0.97 | 3.89 |
| Acetic acid | Merck | ≥99.7 | 15.343 | 5−250 | 0.9990 | 1.74 | 5.02 |
| Lactic acid | Merck | ≥98 | 12.826 | 5−250 | 0.9997 | 1.45 | 4.84 |
| Tartaric acid | Merck | ≥99.5 | 8.589 | 5−250 | 0.9996 | 0.63 | 3.71 |
| Catechol | Merck | 99 | 33.459 | 5−250 | 0.9997 | 1.22 | 4.54 |
| Tyrosol | Merck | ≥98 | 58.252 | 5−250 | 0.9996 | 1.17 | 3.65 |

LOD: limit of detection; LOQ: limit of quantification.

**Table S2.** Genus-level relative abundance of taxa detected in the enriched SLL and MSD consortia by full-length 16S rRNA gene sequencing. Only genera detected at ≥0.1% relative abundance in at least one consortium are shown. Values are expressed as a percentage of classified reads after application of a minimum abundance threshold of 0.01%. –, not detected or below the reporting threshold.

| Genus | Relative abundance (%) | |
| --- | --- | --- |
|  | SLL | MSD |
| *Escherichia* | 90.1 | 18.1 |
| *Proteus* | 0.3 | 52.5 |
| *Wolinella* | 1.3 | 28.2 |
| *Acetoanaerobium* | 5.3 | – |
| *Proteiniclasticum* | 1.4 | – |
| *Enterococcus* | 0.3 | 0.1 |
| *Shigella* | 0.3 | 0.2 |
| *Dethiosulfatibacter* | 0.2 | – |
| *Enterobacter* | – | 0.2 |
| *Macellibacteroides* | 0.2 | – |
| *Proteiniphilum* | – | 0.1 |
| *Anaerospora* | – | 0.1 |
| *Vagococcus* | – | 0.1 |
| *Laceyella* | – | 0.1 |
| *Geovibrio* | 0.1 | – |
| Unclassified/No genus assignment | 0.1 | 0.1 |
